# Knowledge of Head and Neck Cancer Risk Factors and Symptoms: A Cross-Sectional Survey Among Arab Americans

**DOI:** 10.1007/s10903-025-01701-1

**Published:** 2025-05-23

**Authors:** Reem F. Siddiqui, Nada Al-Antary, Marissa Gilbert, Lamis Fakhoury, Milkie Vu, Farzan Siddiqui, Eric Adjei Boakye

**Affiliations:** 1 Department of Radiation Oncology, Henry Ford Health, Detroit, MI USA; 2 Department of Public Health Sciences, Henry Ford Health, 1 Ford Place, Detroit, MI 48202 USA; 3 Department of Preventive Medicine, Feinberg School of Medicine, Northwestern University, Chicago, IL USA; 4 Henry Ford Health + Michigan State University Health Sciences, Detroit, MI USA; 5 Department of Otolaryngology – Head and Neck Surgery, Henry Ford Health, Detroit, MI USA; 6 Department of Epidemiology and Biostatistics, Michigan State University College of Human Medicine, East Lansing, MI USA

**Keywords:** Head and neck cancer, Arab Americans, Risk factors, Symptoms, Knowledge, Michigan

## Abstract

We examined head and neck cancer (HNC) risk factors and symptoms knowledge among Arab Americans in Michigan. A survey of 295 adult Arab Americans (≥ 18 years) was conducted between March and July 2023. Knowledge of HNC risk factors and symptoms were assessed using 10 and 14 questions, respectively. For each question, we asked, “Do you know that the following can be risk factors/symptoms of HNC?” and respondents chose “yes,” “no,” or “don’t know/not sure.” Regarding risk factors- tobacco smoking and chewing were the most recognized risk factors, identified by 78.9% and 72.8%, respectively. Less than half identified human papillomavirus (HPV) (40.6%) as risk factor. Regarding symptom knowledge, only 40–50% identified the most common HNC symptoms: nonhealing ulcers (41%), dysphagia or odynophagia (51.4%), and voice changes (48.6%); while 60.6% identified swelling or lump in neck/throat. Approximately, half of the respondents recognized persistent mass or lesion on the tongue (53.3%) and bleeding in the mouth/throat (50.3%) as symptoms of HNC. Education level, household income, and number of sexual partners were associated with knowledge regarding some common symptoms of HNC. While there is higher knowledge about tobacco as a risk factor for HNC, knowledge about HPV as a risk factor is very low. Similarly, knowledge about HNC symptoms is suboptimal in the Arab American community. Findings support the development of interventions for this community to help improve HNC knowledge and related health outcomes.

**Supplementary Information:**

The online version contains supplementary material available at 10.1007/s10903-025-01701-1.

## Introduction

An estimated 71,100 people will be diagnosed with head and neck cancers (HNC) in the United States (U.S) in 2024 [1]. Previous studies recognized tobacco use, alcohol consumption and human papillomavirus (HPV) as significant HNC risk factors [2, 3]. Moreover, the incidence of HPV-associated oropharyngeal cancers has been steadily increasing over the past three decades in the U.S and globally [4, 5]. HNC present with various symptoms including lump in the neck/mouth, nonhealing ulcers, dysphagia or odynophagia, and voice changes [6]. Early HNC detection (stages I and II) offers high cure rate with 80–90% survival, compared to less than 50% survival in late stages (III and IV) [7]. Unlike other cancers, there are no population-based HNC screening programs, emphasizing the importance of risk factor modification, and early recognition of HNC signs and symptoms to promote prevention strategies.

While data on cancer incidence, risk factors and early symptoms knowledge is well-documented among White and African American populations [8, 9], it is lacking for Arab Americans. Arab Americans have been historically grouped in the same category with non-Hispanic Whites in all federally generated demographic data and census [10]. Due to this lack of data disaggregation, it is difficult to estimate the true number of Arab Americans in the U.S or examine their unique patterns of health knowledge, behaviors, and outcomes. While the census-estimated Arab American population is a little over 2 million, the Arab American Institute suggests a figure closer to 3.7 million, concentrated mainly in California and Michigan. In particular, Dearborn, Michigan has the largest percentage of Arab Americans, with 40% of residents having Arab ancestry [11].

Existing research on Arab Americans, particularly regarding cancer screening and incidence, has focused on individuals living in ethnic enclaves [12]. This is a result of Arab Americans not being identified as a distinct ethnic group by the U.S Census Bureau, coupled with their geographical concentration in specific regions across the country [13]. The U.S. Census Bureau has updated Statistical Policy Directive No. 15 in December 2024 to include individuals of Middle Eastern and North African (MENA) descent as a distinct racial/ethnic category [14]. Not everyone who is of MENA descent identifies as Arab. Conversely, the term Arab American specifically refers to people of Arab descent who live in or are citizens of the United States. Arab-Americans may be from various MENA countries but may also have different cultural experiences due to their American upbringing. There is widespread recognition that Arab Americans need to be studied as a distinct minority group due to their religious, cultural, and ethnic background [15]. This is essential for understanding and improving their health outcomes and addressing health disparities, especially when evaluating social determinants of health, health literacy, healthcare access, and preventive health.

Recent research has focused on health assessment, cancer incidence and smoking behaviors among this population. Previous reports demonstrated high smoking rates and low HPV vaccination rates among some Arab American communities [16, 17]. However, as there is no data currently available on this topic, we examined the knowledge of HNC risk factors and symptoms among Arab Americans in Michigan. As secondary exploratory outcomes, we examine factors that were associated with the most common three risk factors and four symptoms of HNC. Findings from this study will guide development of future educational interventions to improve HNC knowledge among Arab American populations.

## Methods

### Study Design, Data Collection and Study Sample

This study was approved by the Henry Ford Health Institutional Review Board (IRB# 16216–39). A cross-sectional survey was conducted between March and July 2023. Eligibility criteria was: (1) age 18 or older and (2) self-identifying as Arab American. Those with a previous HNC diagnosis were ineligible to participate. Survey questions were adapted from national sources such as the National Cancer Institute and Centers for Disease Control and Prevention, including the Health Information National Trends Survey, National Health Interview Survey, and the Behavioral Risk Factor Surveillance System [18–20]. Survey questions were pilot tested for cultural appropriateness by eight Arab American community members. A paper-based and an electronic survey were developed in English and subsequently translated into Arabic by a professional medical translator, in which both were used according to respondents’ preference.

Study data were managed using REDCap (Research Electronic Data Capture) tool hosted at our institution [21]. Respondents were recruited through various fairs, functions, religious and cultural gatherings for Arab Americans in Michigan. These surveys were primarily conducted at large community events such as prayer gatherings in mosques and picnics in the month of Ramadan. Hence, the respondents were mainly Muslims. A total of 500 eligible individuals were randomly approached by research team members in the study time frame and asked if they wanted to participate in the study or not. The purpose of the study was explained, and surveys (paper-based or online link) were provided to those who expressed interest. Verbal consents were obtained from respondents prior to participating. For respondents who completed the survey on paper in-person, research team members were available to assist the respondents in case any clarification was sought regarding any questions. Participation was noncompulsory, and no compensation was offered. The survey was anonymous and no identifiable information, other than age, was collected. The survey took 10–15 min to complete.

### Measures

Knowledge of HNC risk factors were examined with ten questions. We asked: “Are you aware that head and neck cancer can be caused by the following?” Response options included “yes,” “no,” or “don’t know/not sure” for each of the ten questions. Responses of “yes” indicated knowledge of risk factors, while “no” and “don’t know” indicated lack of knowledge. Similarly, knowledge of HNC symptoms were assessed with 14 questions. We asked: “Are you aware that the following can be symptoms of head and neck cancers?” Respondents selected “yes,” “no,” or “don’t know/not sure” for each of the 14 questions. Responses of “yes” indicated knowledge of symptoms, while “no” and “don’t know” indicated lack of knowledge.

Independent variables included age, gender (female, male), highest level of education (college graduate or higher, some college/community college/vocational school graduate), annual household income in U.S dollars (< 50 K, 50 K–100 K, > 100 K), type of health insurance (private, Medicare, Medicaid, other/none), having a regular healthcare provider (yes/no), number of doctor visit in the past year (0, 1–2, ≥ 3), number of dentist visits in the past year (0, 1–2, ≥ 3), number of oral and vaginal sexual partners (none, 1–2, ≥ 3), and ever use of each of the following: cigarette, e-cigarette, hookah, marijuana, and alcohol (never, yes).

### Statistical Analysis

Descriptive statistics were used to analyze characteristics of respondents. HNC risk factors and symptoms knowledge was compared by sociodemographic and behavioral factors, using chi-square tests for categorical variables and independent samples t-test for continuous variables. Adjusted logistic regressions examined associations between sociodemographic characteristics (age, gender, educational level, income, insurance type, access to regular provider, number of doctor and dentist visits) and behavioral factors (number of oral and vaginal sexual partners, tobacco and alcohol use) and knowledge of three of the most documented HNC risk factors (tobacco use, HPV, and excessive alcohol consumption). Similarly, adjusted logistic regressions estimated associations between sociodemographic and behavioral factors and knowledge of four of the most common HNC symptoms (swelling or lump in the neck/throat, nonhealing ulcers, dysphagia or odynophagia, and voice changes). Analyses were performed using SAS statistical software version 9.4 (SAS Institute Inc, Cary, NC). All statistical analyses were 2-tailed and used p-values < 0.05 to determine statistical significance.

## Results

### Study Respondents’ Sociodemographic Characteristics

Out of 500 surveys that were distributed at the various events, 295 respondents returned the completed questionnaire, yielding a response rate of 59%. Of the 295 respondents (mean age of 37.9 ± 14.6), most were females (60.7%), college graduates or higher (57.6%), had private health insurance (56.7%), and a usual place of receiving care (70.7%) (Table 1). Approximately 84% and 80% had at least one doctor visit and dentist visit in the past year, respectively. The majority had no history of tobacco (81.7%) or alcohol use (91.6%).

**Table 1:** Characteristics of Arab Americans in Michigan included in the study (n = 295)

	N (%)
Age (mean ± SD)	37.9 ± 14.6
**Gender**	
Female	179 (60.7)
**Level of education**	
College graduate or higher	170 (57.6)
Some college/vocational school graduate	64 (21.7)
High school graduate or less	61 (20.7)
**Annual household income**	
< $50,000	91 (32.3)
$50,000-$100,000	94 (33.3)
> $100,000	97 (34.4)
**Health insurance**	
Private	166 (56.7)
Medicare	24 (8.2)
Medicaid	57 (19.4)
None/Other	46 (15.7)
**Has usual place of receiving care**	
Yes	203 (70.7)
**# of doctor visits in past year**	
Zero	47 (16.0)
1–2	157 (53.4)
≥ 3	90 (30.6)
**# of dentist visits in past year**	
Zero	58 (19.9)
1–2	194 (66.4)
≥ 3	40 (13.7)
**# of vaginal sexual partners**	
None	95 (37.7)
1–2	118 (46.8)
≥ 3	39 (15.5)
**# of oral sex partners**	
None	145 (54.9)
1–2	83 (31.4)
≥ 3	36 (13.6)
**Cigarette use**	
Yes	51 (18.3)
**E-Cigarette use**	
Yes	36 (13.0)
**Hookah use**	
Yes	72 (26.0)
**Marijuana use**	
Yes	24 (8.7)
**Alcohol use**	
Yes	23 (8.4)

### HNC Risk Factors Knowledge

**Fig. 1:**
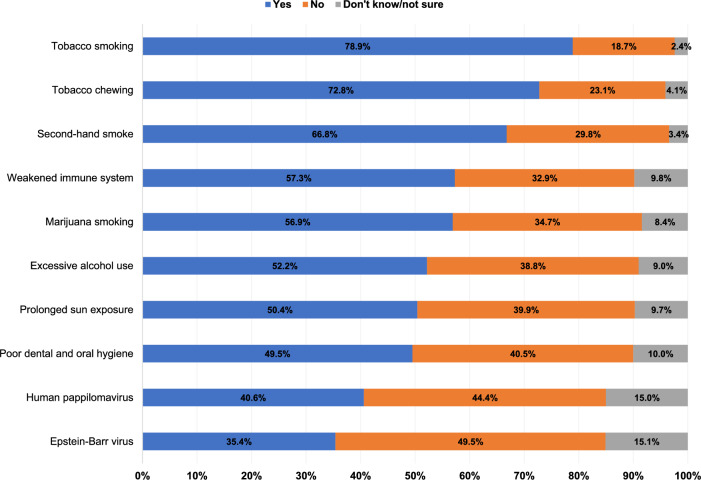
Knowledge of head and neck cancer risk factors among Arab Americans in Michigan (n = 295)

Figure 1 shows study respondents’ knowledge of different HNC risk factors. Tobacco smoking, tobacco chewing, and second-hand smoking were the most recognized risk factors, identified by 78.9%, 72.8% and 66.8%, respectively. However, less than half of the study respondents identified HPV (40.6%) and Epstein-Barr virus (EBV) (35.4%) as HNC risk factors. Moreover, approximately half of the respondents identified excessive alcohol use (52.2%), prolonged sun exposure (50.4%), and poor dental and oral hygiene (49.5%) as HNC risk factors. There were no statistically significant associations between knowledge of the most important HNC risk factors and study respondents’ sociodemographic characteristics, as shown in Table 2.

**Table 2:** Associations between sociodemographic characteristics, behavioral factors, and knowledge of three HNC risk factors (tobacco use, HPV, and excessive alcohol use)

	Adjusted odds ratio (95% confidence interval)		
	^***#***^Tobacco use	^#^HPV	^#^Excessive alcohol use
**Age in years**	0.98 (0.95, 1.02)	1.00 (0.97, 1.02)	1.00 (0.97, 1.02)
**Gender**			
Female	Reference	Reference	Reference
Male	1.41 (0.61, 3.27)	1.10 (0.60, 2.04)	1.40 (0.75, 2.59)
**Level of education**			
College graduate or higher	Reference	Reference	Reference
Some college/vocational sch	0.59 (0.22, 1.60)	1.10 (0.52, 2.33)	1.44 (0.68, 3.07)
High school graduate or less	0.38 (0.13, 1.07)	1.02 (0.44, 2.38)	0.90 (0.39, 2.06)
**Annual household income**			
< $50,000	Reference	Reference	Reference
$50,000-$100,000	0.38 (0.14, 1.06)	0.70 (0.33, 1.48)	0.92 (0.44, 1.94)
> $100,000	0.43 (0.14, 1.33)	1.16 (0.52, 2.58)	0.77 (0.34, 1.73)
**Has usual place of receiving care**			
Yes	Reference	Reference	Reference
No	0.94 (0.40, 2.23)	0.62 (0.32, 1.23)	0.84 (0.44, 1.64)
**# of doctor visits in past year**			
None	Reference	Reference	Reference
≥ 1	0.97 (0.32, 2.90)	0.75 (0.33, 1.71)	1.14 (0.50, 2.60)
**# of dentist visits in past year**			
None	Reference	Reference	Reference
≥ 1	1.95 (0.83, 4.57)	1.42 (0.70, 2.88)	1.02 (0.51, 2.05)
**# of vaginal sexual partners**			
None	Reference	Reference	Reference
≥ 1	2.33 (0.71, 7.71)	1.79 (0.78, 4.15)	2.00 (0.84, 4.73)
**# of oral sex partners**			
None	Reference	Reference	Reference
≥ 1	0.43 (0.15, 1.22)	0.61 (0.29, 1.27)	0.51 (0.24, 1.07)
**Cigarette use**			
Never	Reference	Reference	Reference
Yes	0.87 (0.28, 2.68)	1.05 (0.44, 2.49)	0.62 (0.26, 1.48)
**Alcohol use**			
Never	Reference	Reference	Reference
Yes	0.99 (0.22, 4.49)	0.96 (0.30, 3.10)	1.51 (0.46, 4.98)

^***#***^Each model was adjusted for the following variables: age, gender, educational level, income, insurance type, access to regular provider, number of doctor and dentist visits, number of oral and vaginal sexual partners, tobacco and alcohol use

### HNC Symptoms Knowledge

**Fig. 2:**
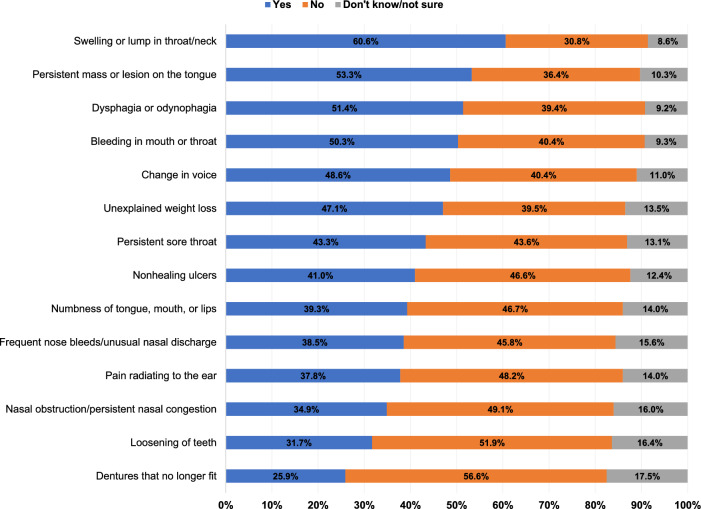
Knowledge of head and neck cancer symptoms among Arab Americans in Michigan (n = 295)

Figure 2 shows study respondents’ knowledge of different HNC symptoms. Only 40–50% identified the most common HNC symptoms; nonhealing ulcers (41%), dysphagia or odynophagia (51.4%), and voice changes (48.6%). Approximately one-third of the respondents reported knowledge of other HNC symptoms including teeth loosening (31.7%), ill-fitting dentures (25.9%), secondary otalgia (i.e., pain radiating to the ear) (37.8%), and nasal obstruction/persistent nasal congestion (34.9%) among others. Meanwhile, swelling or lump in the neck/throat was recognized by 60.6%, followed by persistent mass or lesion on the tongue (53.3%) and bleeding in the mouth or throat (50.3%) as the most common correctly identified HNC symptoms.

Table 3 presents the association between knowledge of the most common HNC symptoms and respondents’ characteristics. Compared with respondents who had college education, those with high school diploma or less had lower odds of identifying nonhealing ulcers (aOR 0.35, 95% CI 0.14, 0.89) or swelling or lump in the neck/throat (aOR 0.38, 95% CI 0.16, 0.92) as a HNC symptoms. Respondents who earn $50 K–$100 K (aOR 0.40, 95% CI 0.18, 0.88) had lower odds of identifying nonhealing ulcers as a HNC symptom compared to those who earn < 50 K annually. Compared with respondents with zero vaginal sexual partners, those with ≥ 1 vaginal sexual partners had a higher odds of identifying nonhealing ulcers (aOR 2.67, 95% CI 1.12, 6.38), dysphagia or odynophagia (aOR 2.74, 95% CI 1.14, 6.56), voice changes (OR 3.37, 95% CI 1.39, 8.17), and swelling or lump in the neck/throat (aOR 3.02, 95% CI 1.18, 7.77) as a HNC symptoms. But respondents with ≥ 1 oral sexual partners (aOR 0.38, 95% CI 0.17, 0.83) had lower odds of identifying voice change as a HNC symptom compared to those with zero partners.

**Table 3:** Associations between sociodemographic characteristics, behavioral factors, and knowledge of four HNC symptoms (swelling or lump in the neck/throat, nonhealing ulcers, dysphagia or odynophagia, and change in voice)

	Adjusted odds ratio (95% confidence interval)			
	^#^Nonhealing ulcers	^#^Dysphagia or odynophagia	^#^Change in voice	^#^Lump in neck or mouth
**Age in years**	0.99 (0.96, 1.01)	0.98 (0.96, 1.01)	0.99 (0.96, 1.01)	0.98 (0.95, 1.01)
**Gender**				
Female	Reference	Reference	Reference	Reference
Male	0.99 (0.53, 1.86)	1.02 (0.55, 1.90)	1.19 (0.64, 2.22)	0.97 (0.50, 1.87)
**Level of education**				
College graduate or higher	Reference	Reference	Reference	Reference
Some college/vocational school graduate	**0.36 (0.16, 0.78)**	0.86 (0.41, 1.83)	0.61 (0.29, 1.29)	0.57 (0.26, 1.26)
High school graduate or less	**0.35 (0.14, 0.89)**	0.77 (0.33, 1.80)	0.51 (0.21, 1.19)	**0.38 (0.16, 0.92)**
**Annual household income**				
< $50,000	Reference	Reference	Reference	Reference
$50,000-$100,000	**0.40 (0.18, 0.88)**	0.53 (0.25, 1.12)	0.69 (0.32, 1.47)	0.57 (0.26, 1.25)
> $100,000	0.48 (0.20, 1.14)	0.80 (0.35, 1.83)	1.07 (0.47, 2.43)	1.02 (0.46, 2.65)
**Has usual place of receiving care**				
Yes	Reference	Reference	Reference	Reference
No	0.71 (0.35, 1.41)	0.62 (0.32, 1.23)	0.94 (0.48, 1.86)	1.12 (0.55, 2.27)
**# of doctor visits in past year**				
None	Reference	Reference	Reference	Reference
≥ 1	0.47 (0.20, 1.09)	**0.39 (0.16, 0.95)**	0.74 (0.32, 1.73)	1.03 (0.43, 2.47)
**# of dentist visits in past year**				
None	Reference	Reference	Reference	Reference
≥ 1	1.28 (0.62, 2.65)	1.06 (0.52, 2.16)	1.40 (0.69, 2.85)	1.53 (0.73, 3.17)
**# of vaginal sexual partners**				
None	Reference	Reference	Reference	Reference
≥ 1	**2.67 (1.12, 6.38)**	**2.74 (1.14, 6.56)**	**3.37 (1.39, 8.17)**	**3.02 (1.18, 7.77)**
**# of oral sex partners**				
None	Reference	Reference	Reference	Reference
≥ 1	0.56 (0.26, 1.20)	0.62 (0.29, 1.32)	**0.38 (0.17, 0.83)**	0.44 (0.19, 1.02)
**Cigarette use**				
Never	Reference	Reference	Reference	Reference
Yes	1.06 (0.44, 2.57)	0.72 (0.30, 1.73)	0.69 (0.29, 1.67)	0.71 (0.28, 1.78)
**Alcohol use**				
Never	Reference	Reference	Reference	Reference
Yes	0.85 (0.26, 2.79)	1.08 (0.32, 3.59)	1.51 (0.45, 5.06)	2.21 (0.55, 8.78)

^***#***^Each model was adjusted for the following variables: age, gender, educational level, income, insurance type, access to regular provider, number of doctor and dentist visits, number of oral and vaginal sexual partners, tobacco and alcohol use

Bold values indicate statistical significance (*P* < 0.05)

## Discussion

The objective of our survey-based study was to assess the knowledge among the Arab American population of Dearborn, Michigan regarding the risk factors and symptoms of head and neck cancers. To our knowledge, this is the first such study done in this population. We noted a high level of knowledge about the traditional etiologic factor of HNC – tobacco smoking (first and second hand) and chewing in this population. However, there was lack of knowledge regarding high-risk HPV as a risk factor. Similarly, EBV was not commonly recognized as an etiologic factor for nasopharyngeal cancers. Furthermore, overall knowledge of the most common symptoms for HNC were low, except for knowledge of swelling or lump in the neck or throat.

Although HPV-associated oropharyngeal cancers are preventable through vaccination against high-risk HPV strains [22], its incidence has been steadily increasing over the past three decades [4]. Our results showed limited knowledge regarding HPV as a risk factor among Arab American respondents. A previous study assessed the knowledge and risk perception of HPV-associated oropharyngeal squamous cell carcinoma (OPSCC) and predictors of oral cancer knowledge in a predominantly African American community and reported lower knowledge and perceived risk of developing HPV-associated OPSCC among Blacks as compared to White individuals [23]. Another study found that Blacks had lower oral cancer knowledge compared to Whites [9]. A focus group conducted among 39 at-risk Hispanics found that none of the participants had knowledge regarding oral cancer and fewer than 30% said that they had heard of oropharyngeal cancer [24]. A national study also found that non-Hispanic Blacks and Hispanics were less likely to know HPV could cause HNC compared with non-Hispanic Whites [25–27]. Promoting HPV vaccination to Arab Americans may pose challenges without concerted efforts to enhance their knowledge of the etiologic role of HPV in HNC. The importance of addressing this issue was further highlighted by a recent study exploring Pap smear outcomes among 125 Arab American women [28]. Among those, 9% were found to have HPV-16 or other high-risk serotypes, suggesting considerable prevalence of HPV circulation in the community, thereby increasing the risk of HPV-related cancers. Long term EBV infection has been associated with a number of malignancies, including those of the oropharynx and nasopharynx [29, 30], which are notably prevalent among individuals of Arab descent or immigrants [31]. EBV being a risk factor for HNC was recognized by approximately one-third of the total survey respondents. Although no vaccinations currently target EBV, educational interventions can enhance individual-level measures taken to avoid different forms of contact with those infected, preventing transmission of primary EBV infection and future consequence contributing to HNC pathogenesis.

We found low overall HNC symptoms knowledge, ranging between 32 and 52%. The level of knowledge presented in this study was lower than that of the general U.S population, where over 55% recognized five of the most common HNC symptoms [32]. Compared to Arab populations outside the U.S, two studies conducted in Saudi Arabia indicated that only 19.4% recognize pain and sore throat as HNC symptoms, while less than 25% recognized red or white patches as early signs of oral cancer, and only 30% were aware that a non-healing ulcer was a symptom of cancer [33, 34]. Meanwhile a study in Poland showed that the most identified symptoms were a lump in the neck (57.9%) and swelling or a lump in the throat (51.8%) [35]. Knowledge of HNC symptoms among Arab Americans remains suboptimal which may result in delayed healthcare seeking and increased risk of advanced stage diagnosis. Future interventional and outreach efforts, particularly targeting those at risk including current or former smokers, is strongly recommended to promote timely seeking of medical attention and early response to HNC symptoms.

Our results show that respondents with one or more vaginal sexual partners had higher odds of identifying the most common HNC symptoms compared to those with zero vaginal sexual partners. While direct evidence linking the number of sexual partners to awareness of HNC symptoms is lacking, this trend might be explained by different theories. Individuals with more sexual partners might have increased awareness of sexually-transmitted infections and HPV, a significant risk factor for HPV-related oropharyngeal cancer. Moreover, people who are sexually active might be exposed to health campaigns that focus on the association between sexual behaviors and different cancers. Further research is needed to capture the different factors contributing to increased HNC symptoms awareness with having one or multiple sexual partners. Moreover, our findings indicate that respondents with at least a college degree or higher income were more likely to recognize non-healing mouth ulcers and neck swelling or lumps as HNC symptoms. These findings align with prior research on cancer screening, showing that higher education, longer residency in the U.S, higher income, and health insurance correlate with better cancer knowledge and outcome [17, 36]. To address disparities, educational interventions should target those with lower socioeconomic status.

Without improved knowledge of HNC risk factors and symptoms, there is a higher chance of increasing HNC morbidity, mortality and health disparities among this population. Culturally relevant interventions such as using peer educators or trusted community leaders and organizations, could be employed to encourage educational campaigns around HNC. Customized cancer education tailored to the Arab American population, including but not limited to Arabic language materials, has been consistently recommended [15, 37, 38]. Future research should develop curriculum and educational programs that could be delivered by HNC experts at community-based events. In addition, future research should use qualitative methodology to elicit what the study population would want and use the feedback to develop educational content in English and Arabic for HNC prevention and early detection.

### Strengths and Limitations

This study has notable strengths. It is the first study to focus on HNC risk factors and symptoms knowledge in an understudied population. Respondents were recruited through community-based venues, which increased the diversity of the sample. Moreover, surveys were provided in both English and Arabic to accommodate those with limited English proficiency. Finally, surveys were administered by researchers who provided “at-the-elbow” support to respondents in case any question was not understood. This study also has some limitations. First, the study was conducted solely in Michigan, which despite having a large Arab American population with many third or fourth generation respondents, these findings may still not be generalizable to all Arab Americans in the U.S. Second, although all respondents self-identified as Arab Americans, they were not separated based on country of origin or ancestry due to many identifying their lineage as mixed from multiple previous generations as well as privacy concerns. There is also the possibility for volunteer bias in our responders. Third, it is still possible that some respondents did not understand some of the terms used in the questionnaire even with at-the-elbow support. Finally, data to differentiate those born in the U.S versus elsewhere was not collected, nor duration of U.S residency. This was excluded due to expected possible hesitation in answering the survey.

## New Contributions to the Literature

In a sample of Arab Americans, we found high knowledge about tobacco use and low knowledge about HPV as important HNC risk factors. Similarly, knowledge about HNC symptoms is suboptimal in the Arab American community, which could result in late-stage presentation and thus lower survival. Future research should focus on creating community centered educational programs in English and Arabic to educate Arab Americans regarding HNC prevention, detection, and treatment.

## Supplementary Information

Below is the link to the electronic supplementary material.

Supplementary file1 (PDF 709 KB)

Supplementary file2 (PDF 169 KB)

## Data Availability

Analytic data will be provided by the corresponding author upon reasonable request.
