# Supplementary material for: Knowledge of Head and Neck Cancer Risk Factors and Symptoms: A Cross-Sectional Survey Among Arab Americans
Source: J Immigr Minor Health. 2025 May 23;27(4):529–38. doi: 10.1007/s10903-025-01701-1 (PMC12255575; doi:10.1007/s10903-025-01701-1)
Supplement: Supplementary file 1 — Supplementary file1 (PDF 709 KB) [file 10903_2025_1701_MOESM1_ESM.pdf]

تاريخ الموافقة
09 مارس 2023
INSTITUTIONAL REVIEW BOARD

APPROVED
April 04, 2023
INSTITUTIONAL REVIEW BOARD

تقييم المعرفة المتعلقة بسرطان الرأس والرقبة بين الأمريكيين ذوي الأصول العربية

تاريخ الموافقة
09 مارس 2023
INSTITUTIONAL REVIEW BOARD

تقييم المعرفة المتعلقة بسرطان الرأس والرقبة بين الأمريكيين ذوي الأصول العربية

رقم التعريف الشخصي : Dr. Eric Adjei Boakye, PhD

الموافقة الطوعية: يطُلب منك المشاركة في دراسة بحثية. وستكون المشاركة طوعية . ولن تواجه عقوبة أو خسارة في حالة اختيارك عدم المشاركة أو التوقف عن المشاركة.

الغرض: الغرض من هذا الاستبيان/هذه الدراسة هو تقييم المعرفة الحالية بسرطان الرأس والرقبة بين الأمريكيين ذوي الأصول العربية، وسيوجه التدخلات المعنية بتحسين النتائج الصحية لهم في المستقبل.

المدة: من المتوقع أن تحتاج 10 دقائق لاستكمال الاستبيان

. الإجراءات والأنشطة:

سيطُلب منك إكمال استبيان موجز يتألف من39 سؤالًا ٠

المخاطر: لا توجد مخاطر أو مضايقات متوقعة نتيجة لمشاركتك في هذه الدراسة. ومع ذلك، قد تجعلك بعض الأسئلة في الاستبيان تشعر بعدم الارتياح. ويمكنك اختيار عدم الإجابة عن أي سؤال تشعر بعدم الارتياح تجاهه.

المزايا: ربما لا تحمل المشاركة في هذه الدراسة إفادة لك بصفة مباشرة. ومع ذلك، قد تفيد مشاركتك الآخرين في المستقبل من خلال

توجيه تطوير التدخلات لتقديم يد العون للسكان الأمريكيين ذوي الأصول العربية.

البدائل: باعتباره أحد البدائل عن المشاركة، يمكنك اختيار عدم المشاركة في هذه الدراسة.

التكاليف والتعويضات: لا يوجد تكاليف ولا تعويضات لمشاركتك في هذه الدراسة.

السريّة : لن تجمع أي معلومات شخصية عند الانتهاء من هذا الاستبيان. وستظل مشاركتك مجهولة.

الأسئلة: إذا كان لديك أي أسئلة إضافية حول إجراءات الدراسة أو للإبلاغ عن إصابة، يمكنك الاتصال بالدكتورDr. Eric Adjei Boakyeعبر البريد الإلكتروني على .eadjei1@hfhs.org إن كنت ترغب في مناقشة حقوقك باعتبارك مشاركًا في البحث، ومناقشة المشكلات والمخاوف والأسئلة أو الحصول على معلومات أو تقديم مدخلات مع شخص مطلع لا ينتمي إلى البحث المحدد، يمكنك الاتصال بمكتب إدارةHenry Ford Health IRB عبر الهاتف على 4464-874-(313) أو عبر البريد الإلكتروني على research_admin@hfhs.orgمجلس المراجعة المؤسسية "(IRB)" هو مجموعة من الأشخاص الذين يقومون بمراجعة البحث لحماية حقوقك.

بيان الموافقة: من خلال إكمال هذا الاستبيان، فإنك تقر بأنك قد وافقت على المشاركة في هذه الدراسة.

(تاريخ الإصدار 01/27/2023)

التركيبة السكانية

1. كم عمرك؟ ______________ سنة

2. ما جنسك؟
   a) ذكر
   b) أنثى
   c) أخرى – حدّد__________________________________

3. ما لغتك المفضلة للرعاية الصحية؟
   a) العربية
   b) الإنجليزية
   c) ايًا منهما

4. ما أعلى درجة علمية حصلت عليها؟
   a) أقل من شهادة الثانوية العامة
   b) دبلومة الثانوية العامة
   c) بعض خريجي الكلية/كلية المجتمع/المدرسة المهنية
   d) خريج كلية أو أعلى

5. ما حالة توظيفك؟
   a) موظف بدوام كامل
   b) موظف بدوام جزئي
   c) عامل لحسابك الخاص
   d) متقاعد
   e) طالب
   f) غير قادر على العمل أو عاجز
   g) عاطل عن العمل

6. ما الدخل السنوي لأسرتك؟
   a) أقل من 25,000 دولار
   b) من 25,000 دولار إلى 34,999 دولارًا
   c) من 35,000 دولار إلى 49,999 دولارًا
   d) من 50,000 دولار إلى 74,999 دولارًا
   e) 75,000 دولار إلى 99,999 دولارًا
   f) من 100,000 دولار إلى 149,999 دولارًا
   g) 150,000 دولار أو أكثر

7. ما نوع التأمين الصحي أو خطط التغطية الصحية التي لديك؟
   a) لا يوجد تأمين
   b) خاص (على سبيل المثال، Blue Cross، HAP، Molina، إلخ)
   c) Medicare
   d) Medicaid
   e) غير ذلك

8. هل يوجد مكان تذهب إليه عادة للرعاية الروتينية/الوقائية؟
a) نعم
b) لا
c) لا أعرف/غير متأكد

9. هل تحتاج مترجمًا فوريًا للحصول على الرعاية الصحية؟
a) نعم
b) لا

10. كم مرة تحتاج إلى شخص لمساعدتك عندما تقرأ التعليمات أو الكتيبات أو المواد المكتوبة الأخرى المُقدمة من طبيبك أو الصيدلية؟
a) أبدًا
b) نادرًا
c) في بعض الأحيان
d) غالبًا
e) دائمًا

11. على مدار الـ 12 شهرًا الماضية، كم مرة ذهبت إلى عيادة الطبيب؟
a) لم أذهب
b) من مرة إلى مرتين
c) من ثلاث إلى خمس مرات
d) ست مرات أو أكثر
e) لا أعرف/غير متأكد

12. على مدار الـ 12 شهرًا الماضية، كم مرة ذهبت إلى عيادة طبيب الأسنان؟
a) لم أذهب
b) من مرة إلى مرتين
c) أكثر من مرتين
d) لا أعرف/غير متأكد

معرفة سرطان الرأس والرقبة

13. هل سمعت في وقت سابق عن سرطان الرأس والرقبة (الفم والحلق واللسان واللوزتين والأنف)؟
a) نعم
b) لا
c) لا أعرف/غير متأكد

14. هل سبق لأي شخص في عائلتك أن أصيب بسرطان الرأس والرقبة (الفم أو الحلق أو اللسان أو اللوزتين أو الأنف)؟
a) نعم
b) لا
c) لا أعرف/غير متأكد

15. هل تعلم أن سرطان الرأس والرقبة يمكن أن يحدث بسبب ما يلي؟

	نعم	لا	لا أعرف أو غير متأكد
تدخين التبغ (السجائر أو السيجار أو الغليون)			
التدخين غير المباشر			
مضغ التبغ			
تدخين الماريجوانا			
فيروس الورم الحليمي البشري (Human Papilloma Virus, HPV)			
فيروس إبشتاين-بار (Epstein-Barr Virus, EBV)			
الشرب المفرط للكحول			
التعرض لأشعة الشمس لمدد زمنية طويلة			
سوء نظافة الأسنان والفم			
ضعف الجهاز المناعي			

16. هل تعلم أن الأعراض التالية يمكن أن تصبح أعراضًا لسرطان الرأس والرقبة؟

	نعم	لا	لا أعرف أو غير متأكد
االقروح الحمراء او البيضاء/ القروح التي لا تلتئم			
ألم مستمر أو صعوبة في البلع			
تغيير في الصوت			
انتفاخ او تورم في الحلق/الرقبة			
وجود كتلة أو آفة مستمرة على اللسان			
نزيف في الفم أو الحلق			
ألم الأذن			
التهاب الحلق المستمر			
ارتخاء الأسنان			
أطقم أسنان لم يعد قياسها مناسب			
خدر اللسان أو الفم أو الشفتين			
انسداد الأنف أو احتقان الأنف المستمر			
نزيف الأنف المتكرر وإفرازات أنفية غير عادية أو أيا مًنهما			
فقدان الوزن دون أسباب			

فحص سرطان الفم

17. هل تحدث طبيب/طبيب أسنان معك في وقت سابق عن سرطان الفم أو الحلق أو اللسان أو اللوزتين أو الأنف؟

a) نعم
b) لا

18. هل سبق أن أوصى طبيب/طبيب أسنان أو أحالك إلى الخضوع لفحص الفم أو الحلق أو اللسان أو اللوزتين أو الأنف؟
a) نعم
b) لا

19. هل خضعت للفحص للكشف عن سرطان الفم أو الحلق أو اللسان أو اللوزتين أو الأنف؟
a) نعم
b) لا

المعرفة بفيروس الورم الحليمي البشري والسرطانات المرتبطة بفيروس الورم الحليمي البشري

20. هل سمعت في وقت سابق عن فيروس الورم الحليمي البشري(HPV) ؟
a) نعم
b) لا
c) لا أعرف/غير متأكد

21. يتوفر لقاح للوقاية من عدوى فيروس الورم الحليمي البشري ويسمى لقاح فيروس الورم الحليمي البشري أو GARDASIL®. هل سبق ان سمعت عن هذا اللقاح؟
a) نعم
b) لا
c) لا أعرف/غير متأكد

22. هل تعلم أن فيروس الورم الحليمي البشري يمكنه التسبب فيما يلي؟

	نعم	لا	لا أعرف أو غير متأكد
سرطان عنق الرحم			
سرطان القضيب			
سرطان الشرج			
سرطان البلعوم (اللسان واللوزة)			
سرطان المهبل			
سرطان الفرج			

حالة التطعيم ضد فيروس الورم الحليمي البشري لنفسك

23. هل سبق لك أن تلقيت حقنة أو لقاح فيروس الورم الحليمي البشري؟
a) نعم --- انتقل إلى السؤال رقم 24
b) لا --- انتقل إلى السؤال رقم 26
c) لا أعرف --- انتقل إلى السؤال رقم27

24. كم عدد حقن فيروس الورم الحليمي البشري التي تلقيتها؟
a) واحد
b) اثنان
c) ثلاثة أو أكثر
d) لا أعرف

25. أي مما يلي يصف على نحو أفضل السبب الرئيسي الذي جعلك تقرر الحصول على اللقاح؟ (يرجى التحقق من كل ما ينطبق)

- ☐ إنه آمن
- ☐ لحمايتي من الإصابة بالسرطان والثآليل التناسلية
- ☐ لحماية شريكي من بعض أنواع السرطان والثآليل التناسلية
- ☐ أنا نشط جنسيا
- ☐ قدمت توصية بهِ من العائلة أو الأصدقاء أو زملاء العمل
- ☐ قدمت توصية بهِ من مقدم الرعاية الصحية الخاص بي
- ☐ سمعت عنها في إعلان تجاري/في الأخبار
- ☐ للحماية من الأمراض المنقولة جنسيًا
- ☐ لحماية نفسي من السرطانات المرتبطة بفيروس الورم الحليمي البشري
- ☐ قام والداي بجعلي/ بالطلب منى الحصول على اللقاح
- ☐ نصحتني ممرضة المدرسة بالحصول على اللقاح
- ☐ احصل دائمًا على التطعيم م
- ☐ غيرذلك ______________________________

26. أي مما يلي يصف على نحو أفضل سبب عدم تلقيك اللقاح؟ (يرجى التحقق من كل ما ينطبق)

☐ خضعت للتطعيم/لا ينطبق عليّ

- ☐ لم أسمع به من قبل /لا أعرف ما يكفي عنه
- ☐ لا أعرف من اين احصل عليه
- ☐ أنا لست نشيطًا جنسيًا
- ☐ لا أستطيع تحمل تكلفة اللقاح
- ☐ الآثار الجانبية المحتملة تقلقني
- ☐ لا أرى حاجتي إليها
- ☐ نقص تغطية الرعاية الصحية
- ☐ لا يوافق شريكي/شخص آخر مهم
- ☐ لم أتلق توصية مقدم الرعاية الصحية بشأن لقاح فيروس الورم الحليمي البشري
- ☐ لدي مخاوف بشأن الآثار الضارة للقاح وسلامته
- ☐ عدم الثقة في نظام الرعاية الصحية
- ☐ عوامل ثقافية
- ☐ أسباب دينية
- ☐ ليس لدي وصول سهل إلى اللقاح
- ☐ غير ذلك ______________________________

27. ما مدى احتمالية حصولك على اللقاح في حالة عرضه أو التوصية به؟

☐ خضعت للتطعيم/لا ينطبق علي

a) غير مرجح للغاية
b) غير مرجح إلى حد ما
c) مرجح إلى حد ما
d) مرجح جدًا
e) مرجح للغاية

حالة تطعيم فيروس الورم الحليمي البشري <u>لأطفالك</u>

28. هل لديك أطفال، أو هل يوجد أي شخص يعيش في منزلك المباشر يتراوح عمره بين 9 و17 عامًا؟

a) نعم --- انتقل إلى السؤال رقم 29
b) لا --- انتقل إلى السؤال رقم 34

29. هل سبق لأطفالك تلقي حقنة أو لقاح فيروس الورم الحليمي البشري

a(نعم --- انتقل إلى السؤال رقم 30
b(لا --- انتقل إلى السؤال رقم 32
c(لا أعرف --- انتقل إلى السؤال رقم 33

30.كم عدد حقن فيروس الورم الحليمي البشري التي تلقاها أطفالك؟

e) واحد
f) اثنان
g) ثلاثة أو أكثر
h) لا أعرف

31.أي مما يلي يصف على نحو أفضل السبب الرئيسي الذي جعلك تقرر تطعيم أطفالك؟ (يرجى التحقق من كل ما ينطبق)

☐ إنه آمن
☐ قدمت توصية به من العائلة أو الأصدقاء أو زملاء العمل
☐ قدمت توصية به من مقدم الرعاية الصحية الخاص بهم
☐ سمعت عنها في إعلان تجاري/في الأخبار
☐ لحمايتهم من الإصابة بالسرطان
☐ للحماية من الأمراض المنقولة جنسياً
☐ احرص دائمًا على تطعيم طفلي/اطفالي
☐ غير ذلك ____________________

32.أي مما يلي يصف على نحو أفضل سبب قرارك بعدم تطعيم أطفالك؟ (يرجى التحقق من كل ما ينطبق)

☐ اطفالي خضعوا للتطعيم
☐ لم أسمع به من قبل /لا أعرف ما يكفي عنه
☐ لا أعرف مكان استلامه
☐ لا أستطيع تحمل تكلفة اللقاح
☐ الآثار الجانبية المحتملة تقلقني
☐ لا أرى حاجتهم اليه
☐ نقص تغطية الرعاية الصحية
☐ لم أتلق توصية مقدم الرعاية الصحية بشأن لقاح فيروس الورم الحليمي البشري
☐ لدي مخاوف بشأن الآثار الضارة للقاح وسلامته
☐ عدم الثقة في نظام الرعاية الصحية
☐ عوامل ثقافية
☐ أسباب دينية
☐ ليس لدي وصول سهل إلى اللقاح
☐ غير ذلك ____________________

33.ما مدى احتمالية السماح لأطفالك على اللقاح في حالة عرضه أو التوصية به؟

☐ اطفالي خضعوا للتطعيم /لا ينطبق على الأطفال
a) غير مرجح للغاية
b) غير مرجح إلى حد ما
c) مرجح إلى حد ما
d) مرجح جدًا
e) مرجح للغاية

عوامل الخطر

34.كم عدد الاشخاص الذين مارست معهم الجنس المهبلي في حياتك؟
a) لم أمارسه
b) ١-٢
c) ٣-٤
d) ٥ أو أكثر

35.كم عدد الاشخاص الذين مارست معهم الجنس الفموي في حياتك ؟
a) لم أمارسه
b) ١-٢
c) ٣-٤
d) ٥ أو أكثر

36.هل لديك تاريخ لأي من العادات التالية؟

	أبدًا	نعم			
		الاستخدام الحالي	محاولة الإقلاع عن التدخين	أقلعت عنه	كم عدد سنوات التدخين؟
هل قمت أو سبق لك تدخين السجائر؟ (باستثناء السجائر الإلكترونية)					
هل قمت أو سبق لك تدخين السجائر الإلكترونية؟					
هل سبق لك تدخين الأرجيلة أو النرجيلة؟					
هل قمت أو سبق لك تدخين الماريوانا					
هل شربت الكحول أو سبق لك تناول الكحول؟					

إدراك مخاطر السرطان

37.ما التصور المتعلق بخطر الإصابة بالسرطان؟

	غير مرجح تمامًا	غير مرجح	ليس مرجح او غير مرجح	مرجح	مرجح جدًا
ما مدى احتمالية إصابتك بالسرطان في حياتك؟					

مقارنة بالأشخاص الآخرين في عمرك، ما مدى احتمالية إصابتك بالسرطان في حياتك؟					
ما مدى احتمالية إصابتك بسرطان **الرأس والرقبة** في حياتك؟					
مقارنة بالأشخاص الآخرين في عمرك، ما مدى احتمالية إصابتك بسرطان **الرأس والرقبة** في حياتك؟					

فرص مستقبلية للتوعية

38. هل أنت على استعداد للمشاركة في جلسة توعية مدتها من 20 إلى 30 دقيقة حول سرطانات الرأس والرقبة يقدمها خبراء في إدارة وعلاج هذه السرطانات؟
   a) نعم
   b) لا
   c) ربما

39. ما الطريقة المفضلة لديك لعرض الجلسة؟
   a) شخصيًا
   b) فيديو مسجل عبر الإنترنت
   c) ندوة مباشرة عبر الإنترنت
