# Supplementary material for: Knowledge of Head and Neck Cancer Risk Factors and Symptoms: A Cross-Sectional Survey Among Arab Americans
Source: J Immigr Minor Health. 2025 May 23;27(4):529–38. doi: 10.1007/s10903-025-01701-1 (PMC12255575; doi:10.1007/s10903-025-01701-1)
Supplement: Supplementary file 2 — Supplementary file2 (PDF 169 KB) [file 10903_2025_1701_MOESM2_ESM.pdf]

APPROVED
March 09, 2023
INSTITUTIONAL REVIEW BOARD

ASSESSMENT OF KNOWLEDGE REGARDING HEAD AND NECK CANCERS AMONG ARAB-AMERICANS

APPROVED
March 09, 2023
INSTITUTIONAL REVIEW BOARD

ASSESSMENT OF KNOWLEDGE REGARDING HEAD AND NECK CANCERS AMONG ARAB-AMERICANS

PI: Dr. Eric Adjei Boakye, PhD

Voluntary Consent: You are being asked to participate in a research study. Participation is voluntary. There will be no penalty or loss of benefits if you choose not to participate or discontinue participation.
Purpose: The purpose of this investigation is to assess the current knowledge of head and neck cancers among Arab-Americans and will guide development of interventions to improve the health outcomes of the Arab American population in the future.
Duration: It is expected that your participation will last for 10 minutes to complete the questionnaire.
Procedures and Activities: You will be asked to complete a brief survey consisting of 39 of questions.
Risks: There are no foreseeable risks or discomforts as a result of your participation in this study. However, some questions in the questionnaire may make you uncomfortable. You may choose not to answer any question with which you feel uncomfortable. Additional risks include a potential breach of confidentiality of your personal information.
Benefits: Participation in this study may not benefit you directly. Your participation may however benefit others in the future by informing interventions development to help the Arab-American population
Alternatives: As an alternative to participation, you could choose to not participate in this study.
Costs and Compensation: There will be no costs and no compensation for your participation in this study.
Confidentiality: No personal information will be collected upon completion of this survey. Your involvement will be kept anonymous.
Questions: If you have any additional questions about the study procedures or to report an injury you may contact Dr. Eric Adjei Boakye by email at eadjei1@hfhs.org. If you would like to discuss your rights as a research participant, discuss problems, concerns, and questions; obtain information; or offer input with an informed individual who is unaffiliated with the specific research, you may contact the Henry Ford Health IRB Administration Office by phone at (313) 874-4464 or by email at research_admin@hfhs.org. The IRB is a group of people who review the research to protect your rights.
Statement of Consent: By completing this survey, you are acknowledging that you have given consent to participate in this study.

(Version Date 1/27/2023)

DEMOGRAPHICS

1. What is your age? ______________ years

2. What is your gender?
   a) Male
   b) Female
   c) Other – Define __________________________________

3. What is your preferred language for healthcare?
   a) Arabic
   b) English
   c) Both

4. What is your highest level of education?
   a) Less than a high school diploma
   b) High school diploma
   c) Some college/community college/vocational school graduate
   d) College graduate or higher

5. What is your employment status?
   a) Full-time
   b) Part-time
   c) Self-employed
   d) Retired
   e) Student
   f) Unable to work or disabled
   g) Unemployed

6. What is your annual household income?
   a) Less than $25,000
   b) $25,000 to $34,999
   c) $35,000 to $49,999
   d) $50,000 to $74,999
   e) $75,000 to $99,999
   f) $100,000 to $149,999
   g) $150,000 or more

7. What type of health insurance or health coverage plans do you have?
   a) No insurance
   b) Private (e.g., Blue Cross, HAP, Molina, etc.)
   c) Medicare
   d) Medicaid
   e) Other
8. Is there a place you usually go for routine/preventive care?
   a) Yes

b) No
c) Don’t know / Not sure

9. Do you use an interpreter for healthcare?
   a) Yes
   b) No

10. How often do you need someone to help you when you read instructions, pamphlets, or other written material from your doctor or pharmacy?
    a) Never
    b) Rarely
    c) Sometimes
    d) Often
    e) Always

11. Over the past 12 months, how many times have you gone to the doctor’s office?
    a) None
    b) 1 – 2
    c) 3 – 5
    d) 6 or more
    e) Don’t know / Not sure

12. Over the past 12 months, how many times have you gone to the dentist's office?
    a) None
    b) 1 – 2
    c) More than 2
    d) Don’t know / Not sure

KNOWLEDGE OF HEAD AND NECK CANCER

13. Have you ever heard of head and neck (mouth, throat, tongue, tonsil, nose) cancer?
    a) Yes
    b) No
    c) Don’t know / Not sure

14. Has anyone in your family ever had head and neck (mouth, throat, tongue, tonsil, or nose) cancer?
    a) Yes
    b) No
    c) Don’t know / Not sure
15. Are you aware that head and neck cancer can be caused by the following?

	YES	NO	DON'T KNOW or NOT SURE
Tobacco (cigarettes, cigars, or pipe) smoking			
Second-hand smoke			
Tobacco chewing			
Marijuana smoking			
Human Papilloma Virus (HPV)			
Epstein-Barr Virus (EBV)			
Excessive alcohol use			
Prolonged sun exposure			
Poor dental and oral hygiene			
Weakened immune system			

16. Are you aware that the following can be symptoms of head and neck cancers?

	YES	NO	DON'T KNOW or NOT SURE
Red or white sore / sores that do not heal			
Persistent pain or difficulty with swallowing			
Change in voice			
Swelling or lump in throat/neck			
Persistent mass or lesion on the tongue			
Bleeding in mouth or throat			
Pain radiating to the ear			
Persistent sore throat			
Loosening of teeth			
Dentures that no longer fit			
Numbness of tongue, mouth, or lips			
Nasal obstruction or persistent nasal congestion			
Frequent nose bleeds and/or unusual nasal discharge			
Unexplained weight loss			

17. Has a doctor/dentist ever talked with you about mouth, throat, tongue, tonsil, or nose cancer?
    a) Yes
    b) No

18. Has a doctor/dentist ever recommended or referred you to get screened for mouth, throat, tongue, tonsil, or nose cancer?
    a) Yes
    b) No

19. Have you been screened for mouth, throat, tongue, tonsil, or nose cancer?
    a) Yes
    b) No

KNOWLEDGE OF HPV and HPV-ASSOCIATED CANCERS

20. Have you ever heard of the Human Papilloma Virus (HPV)?
    a) Yes
    b) No
    c) Don't know / Not sure

21. A vaccine to prevent HPV infection is available and is called the HPV shot, or GARDASIL®. Before today, have you ever heard of the HPV vaccine?
    a) Yes
    b) No
    c) Don't know / Not sure

22. Are you aware that HPV can cause the following?

	YES	NO	DON'T KNOW or NOT SURE
Cervical cancer			
Penile cancer			
Anal cancer			
Oropharynx (tongue and tonsil) cancer			
Vaginal cancer			
Vulvar cancer			

HPV VACCINATION STATUS FOR YOURSELF

23. Have you ever received an HPV shot or vaccine?
    a) Yes --- go to Q24
    b) No --- skip to Q26
    c) Don't know --- skip to Q27

24. How many HPV shots did you receive?
    a) One
    b) Two
    c) Three or more
    d) Don't know

25. Which of the following best describes the main reason you decided to get the vaccine? (Please check all that apply)
    ☐ It is safe
    ☐ To protect me from getting cancer and genital warts
    ☐ To protect my partner from some cancers and genital warts
    ☐ I'm sexually active
    ☐ It was recommended by family or friends or co-workers
    ☐ It was recommended by my healthcare provider
    ☐ I heard about it on a commercial / in the news
    ☐ To protect against sexually transmitted infections
    ☐ To protect myself against HPV related cancers
    ☐ My parents made/asked me to get the vaccine
    ☐ School nurse advised me to get the vaccine
    ☐ Always get vaccinated
    ☐ Other ________________________________

26. Which of the following best describes why you have NOT received the vaccine? (Please check all that apply)
    ☐ Vaccinated / does not apply to me
    ☐ I have never heard of it / I don't know enough about it
    ☐ I don't know where to receive it
    ☐ I am not sexually active
    ☐ I cannot afford it / cost of the vaccine
    ☐ Potential side effects worry me
    ☐ I don't think I need it
    ☐ Lack of health care coverage
    ☐ My partner/significant other does not approve
    ☐ I have not received provider's recommendation for the HPV vaccine
    ☐ I have concerns about the vaccine's adverse effects and safety
    ☐ Distrust of the health care system
    ☐ Cultural factors
    ☐ Religious reasons
    ☐ I don't have easy access to the vaccine
    ☐ Other____________________________________

27. How likely are you to receive the vaccine if offered or recommended?
    ☐ Vaccinated / does not apply to me
    a) Extremely unlikely
    b) Somewhat unlikely

c) Somewhat likely
d) Very likely
e) Extremely likely

HPV VACCINATION STATUS FOR YOUR CHILDREN

28. Do you have children, or is there anyone living in your immediate household between the ages of 9 and 17 years?
    a) Yes --- go to Q29
    b) No --- skip to Q34

29. Have your children ever received an HPV shot or vaccine?
    a) Yes --- go to Q30
    b) No --- skip to Q32
    c) Don't know --- skip to Q33

30. How many HPV shots did your children receive?
    e) One
    f) Two
    g) Three or more
    h) Don't know

31. Which of the following best describes the main reason you decided to get your children vaccinated? (Please check all that apply)
    ☐ It is safe
    ☐ It was recommended by family or friends or co-workers
    ☐ It was recommended by their healthcare provider
    ☐ I heard about it on a commercial / in the news
    ☐ To protect them from getting cancer
    ☐ To protect them from getting sexually transmitted infections
    ☐ Always get child(ren) vaccinated
    ☐ Other ______________________________________

32. Which of the following best describes why you decided NOT to get your children vaccinated? (Please check all that apply)
    ☐ Children vaccinated / does not apply to children
    ☐ I have never heard of it / I don't know enough about it
    ☐ I don't know where to receive it
    ☐ I cannot afford it / cost of the vaccine
    ☐ Potential side effects worry me
    ☐ I don't think they need it
    ☐ Lack of health care coverage
    ☐ I have not received provider's recommendation for the HPV vaccine
    ☐ I have concerns about the vaccine's adverse effects and safety
    ☐ Distrust of the health care system

☐ Cultural factors
☐ Religious reasons
☐ I don't have easy access to the vaccine
☐ Other____

33. How likely are you to allow your children to receive the vaccine if offered or recommended?
   ☐ Children vaccinated / does not apply to children
   a) Extremely unlikely
   b) Somewhat unlikely
   c) Somewhat likely
   d) Very likely
   e) Extremely likely

RISK FACTORS

34. How many vaginal sexual partners have you had in your lifetime?
   a) None
   b) 1-2
   c) 3-4
   d) 5 or more

35. How many oral sex partners have you had in your lifetime?
   a) None
   b) 1-2
   c) 3-4
   d) 5 or more

36. Do you have a history of any of the following habits?

	**Never**	**Yes**			
		Current use	Trying to quit	Have quit	How many years of use?
Do you or have you ever smoked cigarettes? (excluding e-cigarettes)					
Do you or have you ever smoked e-cigarettes?					
Do you or have you ever smoked hookah/ waterpipe?					

Do you or have you ever smoked marijuana?					
Do you or have you ever drank alcohol?					

CANCER RISK PERCEPTION

37. What is perception regarding your risk for developing a cancer?

	Very unlikely	Unlikely	Neither Unlikely nor Likely	Likely	Very likely
How likely are you to get cancer in your lifetime?					
Compared to other people your age, how likely are you to get cancer in your lifetime?					
How likely are you to get **head and neck cancer** in your lifetime?					
Compared to other people your age, how likely are you to get **head and neck cancer** in your lifetime?					

FUTURE EDUCATIONAL OPPORTUNITIES

38. Would you be willing to participate in a 20-30 minute educational session on head and neck cancers provided by experts in the management and treatment of these cancers?
    a) Yes
    b) No
    c) Maybe

39. How you prefer this session be offered?
    a) In-person
    b) Online-recorded video
    c) Online- live Webinar
